# Rapidly-Deposited Polydopamine Coating via High Temperature and Vigorous Stirring: Formation, Characterization and Biofunctional Evaluation

**DOI:** 10.1371/journal.pone.0113087

**Published:** 2014-11-21

**Authors:** Ping Zhou, Yi Deng, Beier Lyu, Ranran Zhang, Hai Zhang, Hongwei Ma, Yalin Lyu, Shicheng Wei

**Affiliations:** 1 Department of Oral and Maxillofacial Surgery, School and Hospital of Stomatology, Peking University, Beijing, China; 2 Center for Biomedical Materials and Tissue Engineering, Academy for Advanced Interdisciplinary Studies, Peking University, Beijing, China; 3 Suzhou Institute of Nano-Tech and Nano-Bionics, Chinese Academy of Sciences, Suzhou, China; 4 Department of Stomatology, Beijing Anzhen Hospital, Capital Medical University, Beijing, China; 5 Department of Restorative Dentistry, School of Dentistry, University of Washington, Washington, United States of America; University of Akron, United States of America

## Abstract

Polydopamine (PDA) coating provides a promising approach for immobilization of biomolecules onto almost all kinds of solid substrates. However, the deposition kinetics of PDA coating as a function of temperature and reaction method is not well elucidated. Since dopamine self-polymerization usually takes a long time, therefore, rapid-formation of PDA film becomes imperative for surface modification of biomaterials and medical devices. In the present study, a practical method for preparation of rapidly-deposited PDA coating was developed using a uniquely designed device, and the kinetics of dopamine self-polymerization was investigated by QCM sensor system. It was found that high temperature and vigorous stirring could dramatically speed up the formation of PDA film on QCM chip surface. Surface characterization, BSA binding study, cell viability assay and antibacterial test demonstrates that the polydopamine coating after polymerization for 30 min by our approach exhibits similar properties to those of 24 h counterpart. The method has a great potential for rapid-deposition of polydopamine films to modify biomaterial surfaces.

## Introduction

Inspired by the composition of adhesive proteins in mussels, Messersmith et al [1] (2007) employed the innate self-polymerization attribute of dopamine to form thin and surface-adherent polydopamine (PDA) film, which later on has been widely applied as a surface modification agent. Polydopamine possesses covalent and non-covalent bonding capabilities for a broad range of organic, inorganic and metallic substrates [2], harboring potential applications in the challenging field of antibacterial [3], antifungal [4], antifouling [5], [6], drug delivery vehicle [7], biosensor [8], cell culture [9], tissue engineering [10]–[12] and so on. However, to author's knowledge, dopamine solution is usually polymerized for at least several hours prior to surface modification/deposition. It is too long from practical point of view and it is crucial to develop a rapid PDA film formation method.

Although PDA coating has been used for numerous years and tremendous effort has been invested in understanding its structure, it has yet to be unambiguously determined. It is known that the process of dopamine-polymerization first involves oxidation of a catechol to a benzoquinone [13] and PDA film is a complex network with free catechol groups available for further chemical surface modification [14]. However, recent research suggests that PDA is a supra-molecular aggregate of monomers rather than a covalent polymer [15].

Despite the wide application and numerous structure analyses for polydopamine coating, much less effort has been focused on factors that would affect the deposition kinetics of polydopamine film, which has become an obstacle for further optimization. Dopamine self-polymerizes to polydopamine usually under slightly alkaline condition, a pH typical of marine environments (2 g·L^−1^ dopamine in 10 mM Tris-HCl, pH = 8.5). The thickness of PDA film is growing as a function of time in 24 h detected by spectroscopic ellipsometry [16]. Zhao, C et al [17] found that addition of oxidizing agents increased the rate of PDA formation under basic conditions. Moreover, the effect of pH and concentration on the deposition kinetics of dopamine solution has also been studied by spectroscopic ellipsometry, AFM [18] and surface plasmon resonance (SPR) [19]. A constant increase in the maximal film mass, thickness and roughness was observed as an augment in the dopamine concentration from 0.1 g·L^−1^ to 10 g·L^−1^, and a pH value of 8.5 was demonstrated to be the best pH for Tris-HCl buffer solution [18], [19]. Furthermore, Xu,Y et al [20] observed that the self-polymerization speed of dopamine grew as temperature increased from 20°C to 60°C. Besides these influence factors, the reaction method could also play a key role in the deposition kinetics of PDA. There is, however, barely any effort devoted in the subject area, which results in inconsistent reaction temperature and time reported in a variety of research articles [21]–[26]. Currently, there are three major dopamine polymerization processes: static, shake, and stir. With regard to the static method, substrates are submerged in dopamine solution for 16 h [10], 18 h [3], overnight [21], [22] or 24 h [12] at room temperature. In shake method, dopamine solutions are shaken to achieve uniform PDA film on the substrate at quite different reaction temperature and time (such as 1 h at ambient temperature [23], 24 h at ambient temperature [24] and 8 h at 30°C [25]). Stir method is popularly used recently, which protects substrates from the particle deposition in the solution [16]. Unfortunately, like static and shake method, the stirring time for dopamine-polymerization is undefined and varies from several hours [26] to 24 h [18]. Undefined conditions in all three methods generate PDA films of various thickness, mass and quality, and make it impossible to compare studies between different labs.

The main aim of this study is to investigate the deposition kinetics of PDA film as a function of temperature and stirring, and to develop a method for rapidly-deposited PDA coating under standard environment (2 g·L^−1^ dopamine in 10 mM Tris-HCl, pH = 8.5).In this investigation, the impact of temperature and reaction method on the deposition kinetics of PDA coating was evaluated by quartz crystal microbalance (QCM) sensor system, which is commonly used to monitor changes in mass at the quartz crystal sensing surface and capable of real-time *in situ* detection in solution [27]. It could be a powerful analytical tool to study the deposition kinetics and properties of PDA coating. In addition, a unique device was designed for preparation of rapidly-deposited PDA film, in which QCM chip substrates were vertically placed in dopamine solution under vigorous stirring (300 r·min^−1^) at 60°C and QCM sensor system was used to monitor the mass change of PDA coating on chip surface. PDA films were characterized using a variety of analytical tools and bio-performance of these films was also evaluated. Results show that using high temperature and vigorous stirring could dramatically speed up dopamine-polymerization and thirty minutes reaction time was found appropriate for surface modification. Our method has demonstrated great potential of being a rapid and practical approach for surface modification of biomaterials and medical devices using self-polymerized PDA coating.

## Materials and Methods

### Materials

Tris (hydroxymethyl) aminomethane (Tris) was purchased from Beijing Chemical Reagents Company (Beijing, China). Dopamine hydrochloride was obtained from Sigma (Missouri, USA). Bovine serum albumin (BSA) fraction V was purchased from Aladdin (Shanghai, China). Commercial pure titanium (Ti), grade 2, was provided from Northwest Institute for Non-ferrous Metal Research (Xi'an, China). Pure polyetheretherketone (PEEK) was purchased from Hua-jun Special Engineering Plastic Products co., LTD (Changzhou, China). Cefotaxime sodium (CS, C_16_H_16_N_5_O_7_S_2_Na) was provided by Amresco Inc. (Ohio, USA).All reagents were of analytical grade. All aqueous solutions were prepared with distilled water.

### Quartz crystal microbalance and chips

The QCM and chips were purchased from Dongwei Biological Technology Co., LTD (Hangzhou, China).The chips were AT-cut planar silicon oxide-coated QCM crystals (14 mm in diameter) with a 5 MHz (4.95 MHz±50 KHz) nominal resonance frequency. Prior to use, all QCM chips were cleaned in an UV/ozone Tip-Cleaner (BioForce Nanosciences, Ames, IA,USA) for 30 min and thoroughly rinsed with distilled water and ethanol for three times, then dried in a stream of nitrogen. Reaction solution was pumped through sensor with QCM chip by a peristaltic pump (BT100K, Baoding, China). The system and reaction solution were conditioned to a required temperature prior to experiment.

The QCM is based on the piezoelectric influence, where a deposited mass is registered as changes in frequency of an oscillating QCM crystal. The adsorbed mass can then be calculated by the Sauerbrey equation [28]: 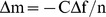
where, Δm equals change in mass per unit surface area, C is the instrument sensitivity constant (17.7 ng·cm^−2^ Hz^−1^), Δf is the frequency change of the specific harmonic and n denotes the number of overtone. The fundamental frequency of our study was simultaneously acquired at of 15 MHz (n = 3).

### The influence of temperature and reaction method on the deposition kinetics and characteristics of polydopamine films

The effect of temperature on the polymerization kinetics of PDA coating was detected *in-situ* by QCM. The clean QCM chip was placed onto the QCM sensor system. QCM sensor system was conditioned at 25°C, 37°C and 60°C respectively prior to experiment. After the baseline became stable, 500 µL 2 g·L^−1^ dopamine solution (10 mM Tris-HCl buffer, pH = 8.5) was placed on the sensor surface to study the influence of temperature on dopamine-polymerization *in situ* and in real-time. Throughout this investigation, all solutions were preheated in a Thermostatic Water Bath (HWSX-650/T, Jinhua, China) at the same temperature to the QCM sensor system.

To investigate the influence of reaction method on dopamine-polymerization, the mass change of chips decorated by PDA films formed by conditions of static, shaking and stirring were measured. For the static method, 3 ml 2 g·L^−1^ dopamine solution (10 mM Tris-HCl buffer, pH = 8.5) was added into each well of 12-well plate (Corning, New York, USA) with cleaned chips and reacted at 60°C for 1 h. In the shaking method, cleaned chips were placed in a 12-well plate. 3 ml standard dopamine solution was injected into each well for 1 h at 60°C with shaking (300 r·min^−1^). In the stirring method, on the other hand, cleaned chips were vertically placed into 200 ml standard dopamine solution at 60°C with stirring (300 r·min^−1^) for 1 h. 300 r·min^−1^ was chosen as the maximal speed that would not affect the vertically placed QCM chips in dopamine solution to investigate the effect of stirring to dopamine-polymerization. The plate and buffer were preheated prior to experiment. After rinsed with distilled water and ethanol, each chip was dried in a stream of nitrogen and the mass change was investigated by QCM sensor system. Each group had three chips and their average values were calculated.

### Preparation of rapidly deposited polydopamine coating

**Figure 1:**
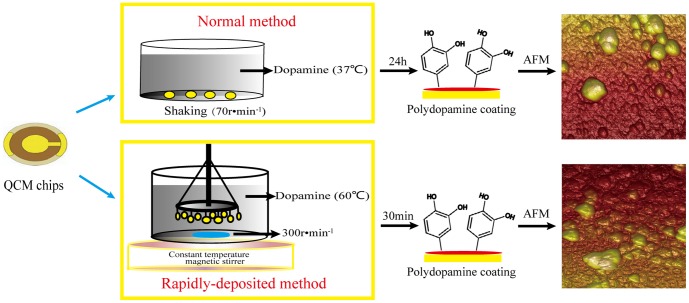
Scheme of polydopamine coating process on QCM chips surfaces by normal method and rapidly-deposited method. Substrates are vertically placed in standard dopamine solution at the temperature of 60°C with stirring (300 r·min^−1^) using our uniquely designed device, which can significantly improve the deposition of polydopamine on QCM chips.

Figure 1 illustrates the formation of polydopamine coating on QCM chips surface by normal method and rapidly-deposited method. In the rapidly-deposited method, 200 ml 10 mM Tris-HCl solution (pH = 8.5) was preheated in a constant temperature magnetic stirrer (SHJ-11, Jinhua, China) at 60°C, and the clean QCM chips were vertically placed into the solution in a uniquely designed device, which was made up of an retort stand, a wire loop and ten small clips. The chips substrates were fixed by the clips with the same interval and their surfaces were in a circle. For PDA coating, 0.4 g dopamine was dissolved and polymerized with vigorous stirring (300 r·min^−1^) for 5 min, 10 min, 20 min,30 min, 1 h, 2 h, 4 h and 8 h respectively. PDA formed by our method named stir-PDA (sPDA). The mass changes of QCM chips decorated by PDA formed at 37°C with gently shaking at the speed of 70 r·min^−1^ for 24 h was studied as the control, named normal method (nPDA). Briefly, the cleaned chips were placed in 12-well plates (Corning, New York, USA). 3 ml 2 g·L^−1^ dopamine solution (10 mM Tris-HCl buffer, pH = 8.5) was injected into each well for 24 h at 37°C with shaking (70 r·min^−1^). The plates and buffer were preheated prior to experiment. After rinsed with distilled water and ethanol, each chip was dried in a stream of nitrogen and the mass change was monitored by QCM sensor. To totally clear away the physically adsorbed PDA particles that not possess covalent bonding capability on substrate surface, PDA coated chips were cleaned in the distilled water by ultrasonic (KQ-5200DE, Kunshan, China) for different periods (1 min, 2 min, 5 min and 10 min) and the mass changes were investigated by QCM.

Moreover, the film thickness of sPDA coated chips with various polymerization times and 24 h-nPDA coated chips that had ultrasonically cleaned for 10 min was further investigated by spectroscopic ellipsometry (MD2000D, J.A.Woollam, Nebraska, USA).

### Characterization

Particles of sPDA and nPDA that acquired from previously reacted dopamine solution were used to evaluate the chemical properties. Briefly, dopamine solutions polymerized by our newly developed method and normal method were filtered by 0.22 um filter respectively, and the obtained PDA particles were dried in a vacuum oven (SDZF-6050, Shanghai, China) at 37°C for 1 h. Fourier-transform infrared spectrometry (FTIR) (Magna-IR 750, Nicolet, USA) was used to identify the functional groups of sPDA and nPDA in the form of pellets (KBr pellet).The spectra were recorded from 4000 cm^−1^ to 400 cm^−1^. The chemical constituents of sPDA and nPDA particles were analyzed by X-ray photo-electronic spectroscopy (XPS) (Kratos, Manchester, UK).The surface hydrophilicity and surface energy (SE) of QCM chips modified by sPDA or nPDA that hat had ultrasonically cleaned for 10 min were characterized by water contact angle measurements (Dataphysics Instrument, Stuttgart, Germany). Three measurements were performed for each chip at ambient temperature based on the sessile drop method, and the mean value was taken as the reported result. The SE value was determined according to OWRK method using distilled water. The surface topographies of sPDA and nPDA films on the surface of QCM chips that had ultrasonically cleaned for 10 min were analyzed by an atomic force microscope (AFM) (Buker, Massachusetts, USA). AFM images were obtained in contact mode. The scan range was 1.0 um×1.0 um, and scan rate was 1 Hz. Ra was used to evaluate the surface roughness on the basis of scan area.Before AFM measurement, the chips were rinsed with distilled water and ethanol, and allowed to air dry.

### BSA immobilization study

BSA was selected as the characteristic protein to investigate the adsorbing properties of sPDA film with various polymerization times (5 min, 10 min, 20 min, 30 min, 1 h, 2 h, 4 h and 8 h), and to compare with that of 24 h nPDA film. Briefly, sPDA and nPDA film decorated QCM chips that had ultrasonically cleaned for 10 min were, respectively, put onto the flow chamber, which was then continuously flushed with 0.01 M PBS buffer (pH = 7.4). After the baseline became stable, the solution was replaced by fresh 5 mg/ml BSA solution in PBS buffer. The reaction was conducted at 25°C by temperature monitor and all used solutions were preheated.

### Cytotoxicity assay

The pristine QCM chips, 30 min-sPDA film coated chips and 24 h-nPDA film coated chips were sterilized under UV irradiation for 30 min, placed in a 24-well plate. The influence of 30 min-sPDA coating and 24 h-nPDA coating on cell viability was evaluated by CCK-8 assay (CCK-8, Dojindo, Kumamoto, Japan), using MG-63 osteoblast cells obtained from the American Type Culture Collection. Cells were grown in Dulbecco's modified Eagle medium (DMEM) (Hyclone, UT,USA), supplemented with 10% fetal bovine serum (Hyclone, UT,USA), 100 U·mL^−1^ penicillin (Amresco, Cleveland, USA), and 0.1 mg·mL^−1^ streptomycin (Amresco, Cleveland, USA) under standard cell culture conditions (37°C, 100% humidity, 95% air and 5% CO_2_).

After cell counting, MG-63 osteoblast cell was seeded into 24-well culture plates with sterilized PDA film modified QCM chips at a density of 2×10^4^ cells/well. The medium was replaced with fresh one every 24 h.

The control experiments were carried out using the complete growth culture medium without substrates (non-toxic control). After incubating for 24 h and 72 h, respectively, 500 µL medium containing 50 µL of CCK-8 reagent was added into each well and incubation went on for two more hours. Then, 200 µL of supernatant from each well was transferred to a new 96-well cell culture plate. The absorbance value (OD value) was measured at 450 nm with a microplate reader (Model 680, Bio-Rad, Hercules, CA). Three specimens were tested for each incubation period, and each test was performed in triplicate.

### Evaluation of antibiotics-binding ability

To study the ability of sPDA film for the immobilization of biomolecules, the inhibition of bacterial adhesion on CS decorated Ti and PEEK surfaces by 30 min-sPDA and 24 h-nPDA was compared. The method for antibacterial activity evaluation is the same as previous reported [29]. Briefly, prior to surface modification, Ti and PEEK were cut into discs of 15 mm in diameter and 2 mm in thickness. These disc samples were polished with a series of SiC abrasive papers (400, 1000, 1500 and 2000 grit). They were then ultrasonically cleaned in acetone, anhydrous ethanol and distilled water, respectively, for 10 min and dried in a stream of nitrogen. 30 min-sPDA coated Ti and PEEK, 24 h-nPDA coated Ti and PEEK were prepared as described previously. 3 ml fresh 50 mg·mL^−1^ CS in distilled water was injected into each well of 12-well plate with modified Ti and PEEK at 37°C with shaking (70 r·min^−1^) for 24 h. They were then rinsed with distilled water and dried in a stream of nitrogen. 30 min-sPDA-CS decorated Ti, 24 h-nPDA-CS decorated Ti, 30 min-sPDA-CS decorated PEEK and 24 h-nPDA-CS decorated PEEK were sterilized under UV irradiation for 30 min on each side. *Escherichia coli* (*E. Coli* 1.1369, obtained from China General Microbiological Culture Collection Center, China) and *Streptococcus mutans* (*S. Mutans* UA159, obtained from American type Culture Collection, USA) were selected as the experimental strains. *E.Coli* and *S.Mutans* were cultured in Luria-Bertani (LB) medium and Brian Heart Infusion (BHI) medium respectively. Bacterial adhesion on the surfaces of pristine Ti, pristine PEEK, 30 min-sPDA-CS modified Ti, 30 min-sPDA-CS modified PEEK, 24 h-nPDA-CS modified Ti and 24 h-nPDA-CS modified PEEK for various period (4 h, 24 h and 72 h) was assessed *via* Microbial Viability Assay Kit-WST (Dojindo, Kumamoto, Japan) and a microplate reader (Elx808, Bio-tek, Vermont, USA).Moreover, the activity of bacterial in the medium was also measured to evaluate the release action of CS on those samples. Minimum bactericidal concentration of CS was added into the mixture of bacterial solution and culture medium as the control.

### Statistical analysis

The results were expressed as mean ± standard deviations (SD) derived from experiment and assessed statistically using Student's t-test. Statistical analysis was carried out with software SPSS13.0 for Windows. Statistical significance was accepted at p<0.05.

## Results and Discussion

### Dopamine-polymerization as a function of temperature and reaction method

**Figure 2:**
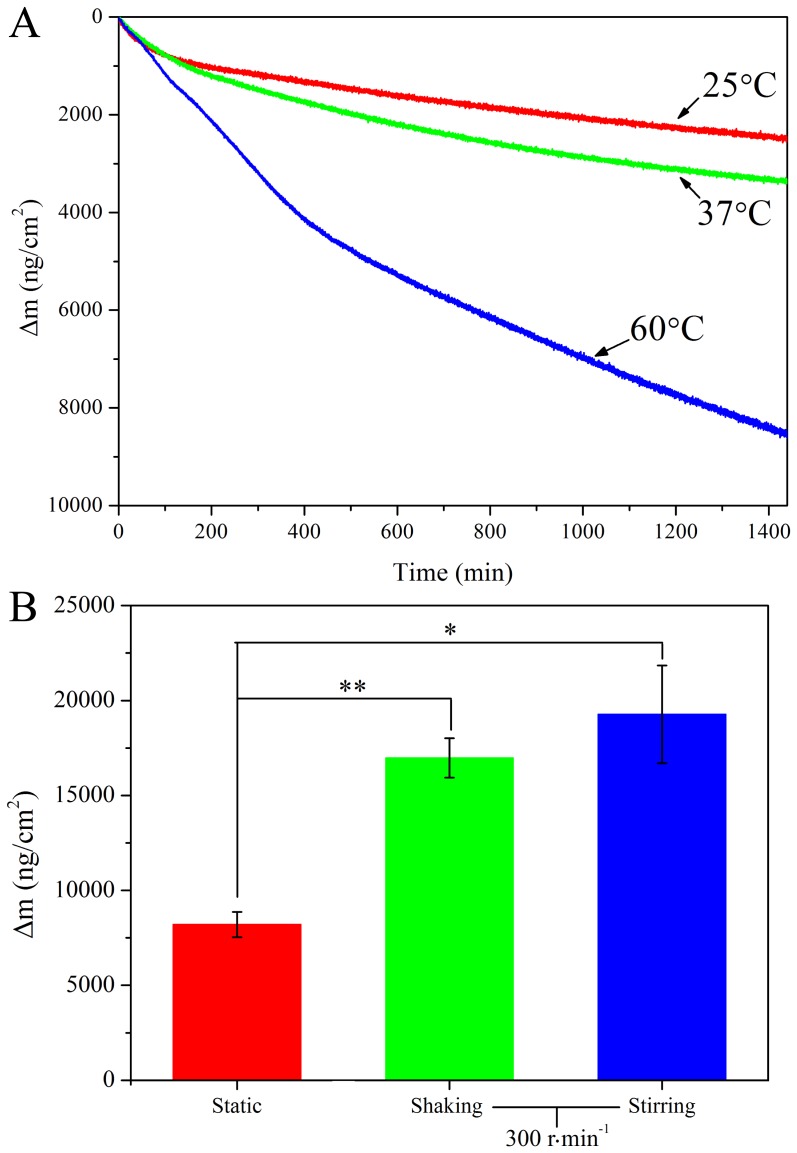
The influence of temperature and reaction method on the deposition kinetics of polydopamine films. (A): Representative mass change vs. time (min) curves as a function of temperature for polymerization of dopamine solution. (B): The mass change of QCM chips coated by PDA formed by methods of static, shaking (300 r·min^−1^) and stirring (300 r·min^−1^) for 1 h respectively. *Represents p<0.05 and ** Represents p<0.01 compared with chips coated by PDA formed at static group, n = 3.

The influence of temperature on the deposition kinetics of PDA coating was *in-situ* and real-time monitored for the first time by QCM (Figure 2A). The Δm signals of PDA films on the sensor surface markedly augment with an increase in the polymerization temperature between 25°C and 60°C, indicating temperature has significant impact on dopamine-polymerization and subsequent deposition. According to the thermodynamic principle, higher temperature would promote the oxidation of dopamine as well as the deposition process of PDA onto substrate surface. This result is consistent with investigation of dopamine polymerization under various temperatures measured by spectroscopic ellipsometry [20].

The mass change of QCM chips decorated by PDA films formed from methods of static, shaking and stirring was measured to evaluate the effect of reaction method on the polymerization behavior of PDA coating (Figure 2B). The mass change of chips decorated by PDA film polymerized for 1 h at 60°C with stirring method (19275±2575 ng·cm^−2^) is a little higher than that of PDA film from shaking method (16981±1037 ng·cm^−2^) (p>0.05), and both are significantly higher than that of PDA film formed at static condition (8202±665 ng·cm^−2^) (p<0.05), indicating vigorous shaking and stirring could dramatically increase the formation speed of PDA film on the sensor surface. That is presumably due to more fresh oxygen in air dissolved in solution with shaking or stirring, which contributes to the self-oxidation of dopamine. However, some disadvantages, such as solution will be splashed out and the chips may turned over to the back side, were observed for dopamine-polymerization with vigorous shaking. This may be the reason why most reported papers we know had used shaking method at low speed for the polymerization of dopamine [23], [25], [29]. Therefore, vigorous stirring possess good potential to be the reaction method to facilitate the polymerization of dopamine solution.

### Mass and thickness of polydopamine film

For surface modification, dopamine solution is generally polymerized for several hours in the presence of substrates, which is too long for any practical applications. Knowing that both high temperature and vigorous stirring could promote the polymerization of dopamine, a unique device was designed, which uses a constant temperature magnetic stirrer for the formation of rapidly-deposited PDA coating. The mass change of chips decorated by sPDA films with varying polymerization times (5 min, 10 min, 20 min,30 min,1 h, 2 h, 4 h and 8 h) was measured by QCM. To better evaluate PDA coating formed by our method, dopamine solution polymerization under normal condition was conducted as control. As mentioned in the introduction, the dopamine-polymerization process is quite different in a variety of research articles [21]–[26]. In our study, condition of 37°C with gently shaking at the speed of 70 r·min^−1^ for 24 h was used as the normal methodfor the reasons as follows: 1). 37°C is the most used reaction temperature; 2). Gentle shaking is popularly applied for the polymerization of dopamine solution [23], [25], [29]; 3). 24 h is the longest reported reaction time reported for PDA coating at which the mass and film thickness will get equilibrium [1].

**Figure 3:**
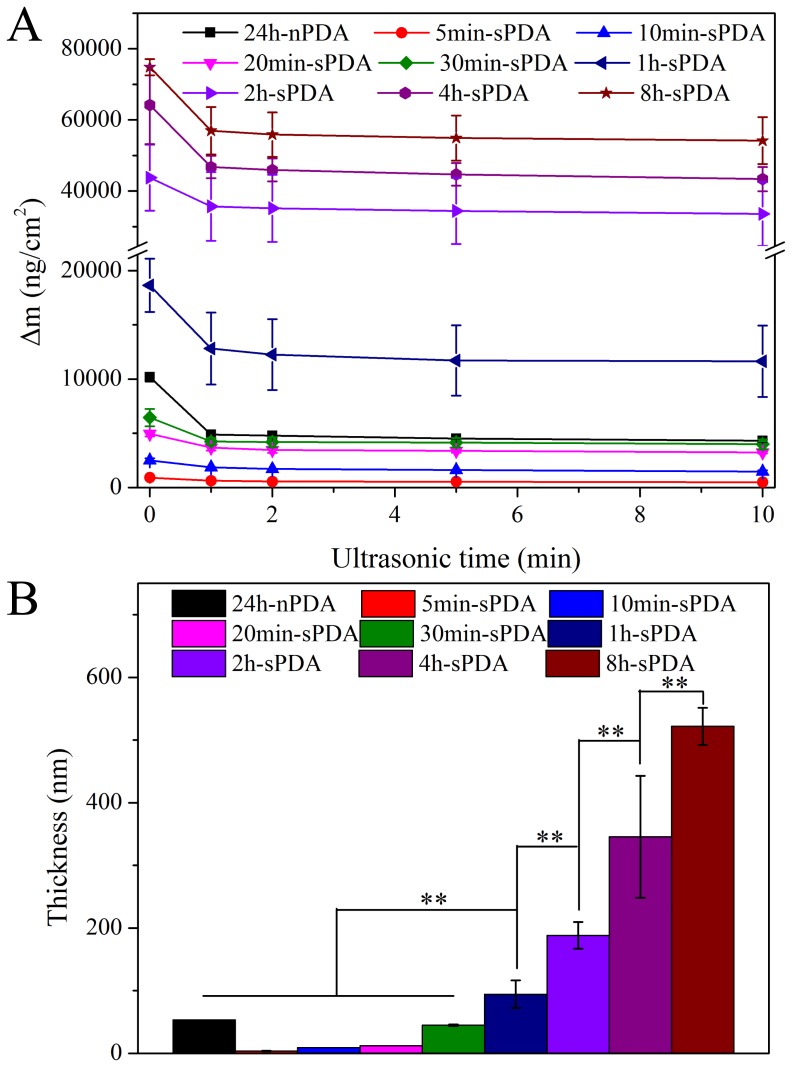
The mass and thickness measurement of sPDA films and nPDA films. (A): The mass change of QCM chips decorated by sPDA films of varying polymerization times (5 min, 10 min, 20 min, 30 min, 1 h, 2 h, 4 h and 8 h) as a function of ultrasonic cleaning time, and compared with that of 24 h-nPDA film coated chips. (B): The film thickness of QCM chips coated by sPDA of different polymerization times (5 min, 10 min, 20 min, 30 min, 1 h, 2 h, 4 h and 8 h) and 24 h-nPDA. Those chips had ultrasonically cleaned in distilled water for 10 min before measurement. ** Represents p<0.01, n = 3.

It has been recognized that excess physically adsorbed PDA particles on the surface of substrate do not have the covalent bonding capability. It is crucial to eliminate the potentially detrimental effect from physically adsorbed PDA particles on the cell functions. PDA films on the substrates are usually washed with distilled water for three times [30], [31]. Compared with conventional water rinsing procedure, ultrasonic cleaning may be more efficient and stable. Unfortunately, various ultrasonic times, such as 5 min [32],10 min thrice [12], [33] and 15 min [34], were reported to clean out physically adsorbed PDA particles on substrate surface, and the cleaning efficiency had not been clearly demonstrated. In the present study, we assessed the mass changes of PDA film decorated chips undergone ultrasonic cleaning for 1 min, 2 min, 5 min and 10 min, respectively. A great amount of physically adsorbed PDA was obliterated after ultrasonic cleaning for 5 min, and mass change of PDA-coated chips reached equilibrium with the extension of time (Figure 3A), suggesting that 5 min treatment is enough to get rid of excessive PDA particles on the sensor surface. This is in consistent with the ultrasonic cleaning time on the surface of silicon wafers reported by Junfei Ou et al [32].

After ultrasonically cleaned, it was found that the mass change of chips modified by PDA coating attained by our method was dramatically increased with the augment of the polymerization time, and reached a plateau after reacted for 8 h (Figure S1 in File S1). After polymerization for 1 h, the mass change of chips decorated by sPDA films is much bigger than that of 24 h-nPDA (p<0.01). Interestingly, the mass change of 30 min-sPDA coated chips (6448±788 ng·cm^−2^) was much less than that of 24 h-nPDA (10186±453 ng·cm^−2^) prior to cleaning (Figure 3A). However, after ultrasonically cleaned, the mass change of chips modified by 30 min-sPDA films (3992±454 ng·cm^−2^) is in close vicinity to that of 24 h-nPDA (4324±178 ng·cm^−2^) (Figure 3A). These results indicate fewer physically absorbed sPDA particles existed on the chip surface than that of nPDA, since vigorous stirring could protect substrates from the particle aggregation in dopamine solution. Besides, there is no PDA on the surface of substrate at the first 30 min by common method [35], But evident mass change was observed when dopamine-polymerization went on for 5 min by our method.

The film thickness of chips coated by sPDA films with different polymerization times (5 min, 10 min, 20 min,30 min,1 h, 2 h, 4 h and 8 h) and 24 h-nPDA that had been ultrasonically cleaned for 10 min was further investigated by spectroscopic ellipsometry. In accordance with the mass change, the film thickness of sPDA on the sensor surfaces increases with the increase in polymerization time and get equilibrium after reacted for 8 h (Figure S1 in File S1). As shown in Figure 3, part B, the thickness of 30 min-sPDA film (44.95±1.15 nm) on the surface of chips is slightly under that of 24 h-nPDA (53.55±0.25 nm) (p>0.05), which is close to 50 nm that reported by Messersmith et al [1]. However, the thickness of sPDA films after polymerized for 1 h are much higher than that of 24 h-nPDA (p<0.01) (Figure 3B). As shown in Figure S3 in File S1, a good correlation was observed for the curve of sPDA mass versus time and that thickness of versus time, indicating the average molecular weight and density of PDA exhibit no significant difference with the augment of polymerization time, since PDA particles is presumed to be hierarchical aggregation comprised with plate-like aggregates at a size of 1–2 nm through π–π stacking [36].

Those mass and film thickness measurements of PDA coating prove that our method using thermal-dynamic treatment of high temperature and vigorous stirring could not only dramatically promote the polymerization speed of dopamine solution, but also deposit much more PDA onto the substrate surface. Moreover, the mass and film thickness of PDA coating after polymerization for 30 min by our approach was similar to those of 24 h counterpart, possess good prospects to be the condition for rapid-deposition of PDA films to modify biomaterial surfaces.

### Characterization of PDA films

**Figure 4:**
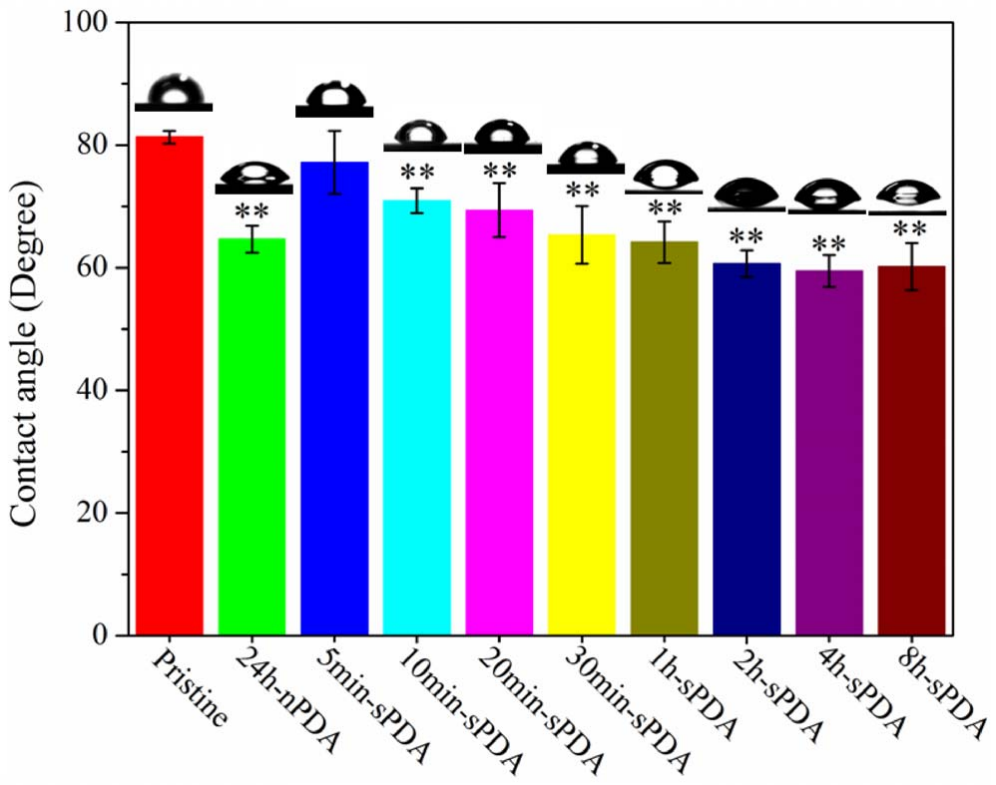
Water contact angles of QCM chips modified by sPDA films and nPDA films. The Contact angles of pristine QCM chips and QCM chips modified by coating of 24 h-nPDA, 5 min-sPDA, 10 min-sPDA, 20 min-sPDA 30 min-sPDA, 1 h-sPDA, 2 h-sPDA, 4 h-sPDA and 8 h-sPDA. All samples were ultrasonically cleaned for 10 min and dried. ** Represents p<0.01 compared with the pristine chips group, n = 3.

The chemical attributes of sPDA and nPDA were probed using Fourier-transform infrared spectrometry (FTIR), X-ray photo-electronic spectroscopy (XPS) and water contact angle measurement. FTIR spectra (KBr) of sPDA and nPDA powders are similar (Figure S2 in File S1). According to Daniel R. Dreyer et al's previous study [15], the peaks at 1510 cm^−1^ and 1600 cm^−1^ are consistent with the indole or indoline structures. The large peak at 3376 cm^−1^ is in accordance with the presence of hydroxyl structures as well as water. No evident difference in chemical element content of C, N and O was observed between sPDA powder and nPDA powder in X-ray photo-electronic spectroscopy (XPS) (Figure S3A in File S1).Moreover, the high-resolution C 1 s spectra of sPDA powder and nPDA powder were deconvoluted into five different curves respectively and they are similar(Figure S3B in File S1). The binding energies centered at 284.60 eV, 285.59 eV, 286.01 eV,287.71 eV and 291.43 eV were assigned to the carbon skeleton (-C-C-/-C-H-), amino group (-C-N-), hydroxyl group (-C-OH), carbonyl group (-C = O and -C(O)O-) respectively [29], [37].These results indicate that rapidly-deposited PDA film formed by the treatment of high temperature and vigorous stirring, compared with nPDA, exhibits no chemical alteration. Figure 4 shows the water contact angles of the pristine QCM chip, sPDA film modified chips with different polymerization times and 24 h-nPDA film modified chips that hat had ultrasonically cleaned for 10 min. The pristine chip is hydrophobic, with a contact angle of 81.30±1.02°. After coated by 5 min-sPDA film, the contact angle of chips presents no evident change. However, significant decrease was observed for the contact angle of chips (70.95±2.03°) when the dopamine-polymerization time increased to 10 min. It remained in the close vicinity to those of 30 min-sPDA (65.34±4.96°), 1 h-sPDA film (64.18±3.40°)and 24 h-nPDA film coated chips (64.67±2.19°). The contact angles reduced to 60.66±2.18° when polymerization time increased to 2 h and reached an equilibrium plateau with the extension of time. These results indicate sPDA coating possesses good hydrophilicity.

**Figure 5:**
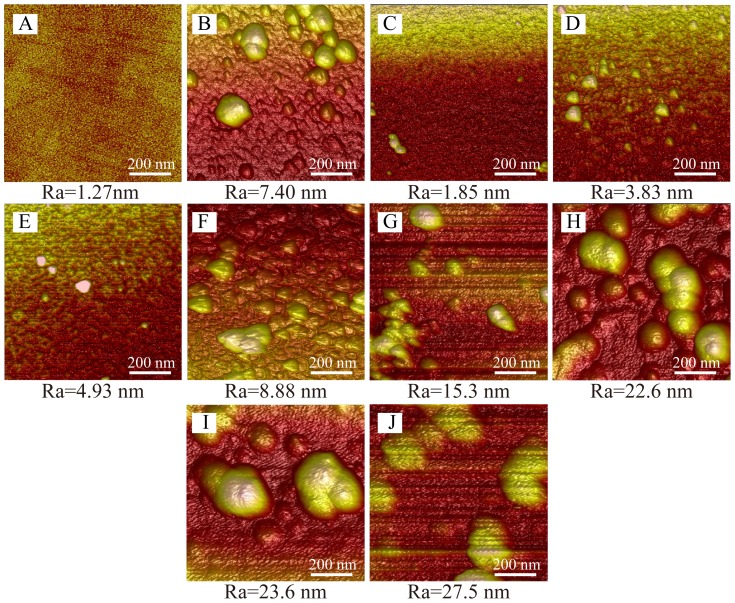
The AFM surface topographies of QCM chips modified by sPDA films and nPDA films. (A): Pristine chip. (B) 24 h-nPDA decorated chip. (C): 5 min-sPDA decorated chip. (D): 10 min-sPDA decorated chip. (E): 20 min-sPDA decorated chip. (F): 30 min-sPDA decorated chip. (G): 1 h-sPDA decorated chip. (H): 2 h-sPDA decorated chip. (I): 4 h-sPDA decorated chip. (J): 8 h-sPDA decorated chip. All those samples had ultrasonically cleaned in distilled water for 10 min. Scale bar: 200 nm.

The surface topographies of sPDA films of varying polymerization times (5 min, 10 min, 20 min,30 min,1 h, 2 h, 4 h and 8 h) and 24 h-nPDA films on chips that had ultrasonically cleaned for 10 min were analyzed by an atomic force microscope (AFM) (Figure 5). The Ra value of chip decorated by sPDA coating augments with the increase of polymerization time. For sPDA coating at 5 min of dopamine polymerization time by our method, partial aggregates were formed on the surface of QCM chips (Figure 5C). When the polymerization time increased to 10 min and 20 min, more PDA aggregates appeared on chip surface, but there was no significant change in particle size and Ra value, which is consistent with the thickness measurement (Figure 5D–E). The surface topography of 30 min-sPDA film modified chip (Ra = 8.88 nm) (Figure 5F) is similar to 24 h-nPDA film coated chip (Ra = 7.40 nm) (Figure 5B), with a significant higher roughness than that of 20 min-sPDA film (Ra = 4.93 nm). Much higher surface roughness is observed for chip coated by sPDA after polymerized for 1 h and the Ra value reaches up to 27.5 nm for 8 h-sPDA (Figure 5G–J). These results are in accordance with the mass change and film thickness measurements, and further confirms that high temperature and vigorous stirring could dramatically improve the polymerization speed of dopamine solution.

### BSA binding ability evaluation

**Figure 6:**
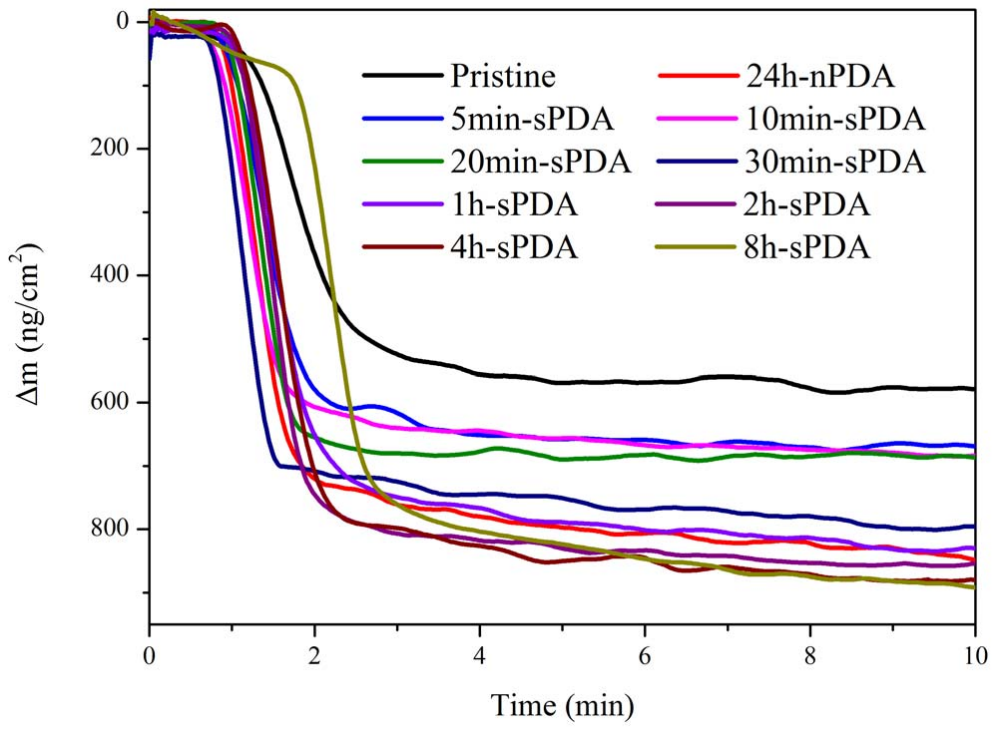
BSA binding evaluation of sPDA films and nPDA films. Representative mass change vs. time (min) curves of pristine chip, 24 h-nPDA coated chip, 5 min-sPDA coated chip, 10 min-sPDA coated chip, 20 min-sPDA coated chip, 30 min-sPDA coated chip, 1 h-sPDA coated chip, 2 h-sPDA coated chip, 4 h-sPDA coated chip and 8 h-sPDA coated chip reaction with 5 mg·ml^−1^ BSA solution.

BSA was selected to be the characteristic protein in real-time evaluation of the protein adhesion properties on the sPDA film coated chips surface by QCM, and in comparison with that of 24 h-nPDA film coated chips (Figure 6). In accordance with the mass measurement, the Δm signals of sPDA films reaction with 5 mg·ml^−1^ BSA solution augment with an increase in polymerization time between 5 min and 8 h, and present higher mass change than that of the pristine chips. The curves of mass change versus time for chips coated by 5 min-sPDA, 10 min-sPDA, 20 min-sPDA reacted with BSA are similar. When the dopamine polymerization time of our method increases to 30 min, much more BSA is bonded onto the chip surface, and the Δm reaches up to 800 ng·cm^−2^, which is slightly less than that of 24 h-nPDA decorated chip (840 ng·cm^−2^).The curve of mass change versus time for chip coated by 1 h-sPDA reacted with BSA is almost overlap with that of 24 h-nPDA. The mass changes of chips coated by 2 h-sPDA (859 ng·cm^−2^), 4 h-sPDA (876 ng·cm^−2^) and 8 h-sPDA (891 ng·cm^−2^) reacted with BSA are similar. The small difference among sPDA coating at a time scale of twenty minutes in film thickness and surface topography contributes to the slow growth of Δm signals reacted with BSA solution. In the same way, the film thickness and surface topography is significantly changed after polymerized for 30 min and a much higher Δm signal was detected. Even though evident augment was observed for the mass changes and film thickness of chips coated by sPDA with the polymerization time increased from 1 h to 8 h, their BSA absorbing ability possess no big difference. The reason is that the surface of sPDA decorated chips has been covered with PDA film after polymerized for 30 min, and there is no significant change in surface coverage with the extension of reaction time.

### Cytotoxicity assay

Previous results indicate coating of sPDA and nPDA exhibit same chemical property. The mass change, film thickness and BSA binding ability of 30 min-sPDA coating is in close vicinity to that of 24 h-nPDA. Moreover, 30 min-sPDA coating and 24 h-nPDA possess similar surface morphology and hydrophilicity. Therefore, 30 min was chosen as the standard polymerization time for our rapidly-deposited PDA coating in follow-up cytotoxicity study and surface functional modification.

**Figure 7:**
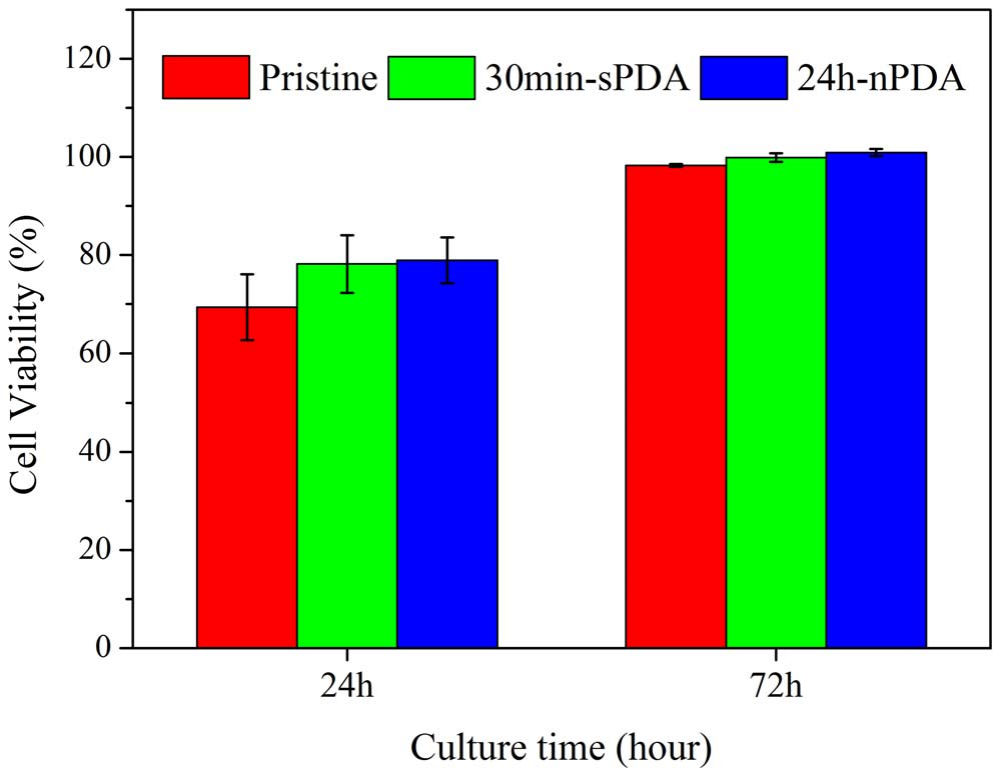
Cytotoxicity assay of QCM chips modified by 30 min-sPDA films and 24 h-nPDA films. Viability of MG63 osteoblasts incubated with pristine QCM chips, 30 min-sPDA decorated chips and 24 h-nPDA decorated chips for 24 h and 72 h. Viability is expressed as a percentage relative to the result obtained with the non-toxic control (MG63 osteoblasts incubated without substrates). n = 3.

The effect of 30 min-sPDA film and 24 h-nPDA film on cell viability was evaluated by CCK-8 assay with MG-63 osteoblasts (Figure 7). After incubated for 24 h, the cell viability of 30 min-sPDA coated chips is slightly higher than that of pristine chips, and similar to that of 24 h-nPDA coated chips. The cell viability of all samples is significantly lower than that of the culture plate (p<0.01). However, MG63 osteoblasts on all samples exhibit almost the same cell viability when incubated for 72 h. Our study suggest that both sPDA film and nPDA film possess good cytocompatibility, which is consistent with previous report [25].

### Antibiotics evaluation

**Figure 8:**
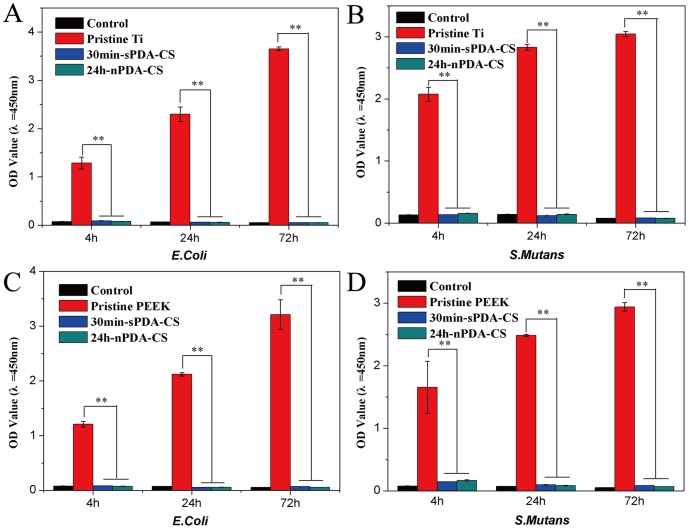
Bacterial adhesion on the surface of 30 min-sPDA-CS and 24 h-nPDA-CS decorated Ti and PEEK. Number of living *E.Coli* and *S.Mutans* adhered on decorated Ti and PEEK surfaces after exposed to bacterial suspension for 4 h, 24 h and 72 h. ** represents p<0.01 compared with the pristine group, n = 3.

Antibiotic property is one of the most important properties for biomaterials, especially for implant applications. In order to explore the practical use of our newly-developed method, inhibition of bacterial adhesion on the surface of 30 min-sPDA-CS and 24 h-nPDA-CS decorated Ti and PEEK for up to 72 h was investigated and the results are shown in Figure 8. For the amount of adhered viable *E.Coli* and *S.Mutans*, no statistically significant difference was found between 30 min-sPDA-CS modified Ti and 24 h-nPDA-CS modified Ti for 4 h, 24 h and 72 h (p>0.05). Moreover, their bacteria adhesion amounts are dramatically lower than that of the pristine Ti (p<0.01) (Figure 8A–B). Inhibition of bacterial adhesion on the surface of 30 min-sPDA-CS decorated PEEK and 24 h-nPDA-CS decorated PEEK, on the other hand, is the same to that of Ti (Figure 8C–D). Furthermore, both for Ti and PEEK, the number of *E.Coli* and *S.Mutans* in the medium with 30 min-sPDA-CS modified substrates is similar to that of 24 h-nPDA-CS modified substrates for up to 72 h (p>0.05), which is significantly lower than that of the pristine (p<0.01) (Figure S4 in File S1). These results demonstrate superior bioactivity of PDA films formed by our method, which has great potential to be universal approach for surface modification.

## Conclusions

In this study, a new method for the formation of rapidly-deposited PDA film was developed, in which substrates are vertically placed in standard dopamine solution at the temperature of 60°C with vigorous stirring (300 r·min^−1^) using an uniquely designed device. The influence of temperature and stirring on the deposition kinetics of PDA film was investigated by the quartz crystal microbalance (QCM). Our results indicate that high temperature and vigorous stirring both can dramatically speed up the rate of dopamine-polymerization. Physical treatment of high temperature and vigorous stirring would result in no chemical changes for polydopamine. The mass, thickness and BSA binding ability of PDA film formed by our approach for 30 min are comparable to those of shaking counterpart for 24 h. Moreover, cell viability assay and antibacterial test demonstrate that our rapidly-deposited PDA coating possesses equally excellent surface modification properties compared with PDA formed by normal method. Furthermore, substrates placed vertically in our approach could not only reduce the deposited PDA aggregates from solution, but also be decorated with PDA film on all surfaces. Our method for rapid deposition of polydopamine film may be used as a potential approach for the surface modification of biomaterials and medical devices.

## Supporting Information

File S1 Contains the following files: Figure S1, Mass change versus time and film thickness versus time for sPDA decorated chips. The black curve shows the mass changes versus time of QCM chips coated by sPDA with various polymerization time (5 min, 10 min, 20 min, 30 min, 1 h, 2 h, 4 h and 8 h) that had ultrasonically cleaned for 10 min. The red curve shows the film thickness versus time of the same samples. n = 3. Figure S2, FTIR spectra analysis. The FTIR spectra (KBr) of nPDA powder and sPDA powder. Figure S3, XPS survey scan spectra of nPDA powder and sPDA powder. (A): XPS wide spectra of nPDA powder and sPDA powder, insert table shows the contents of C, N and O. (B): High-resolution spectrum of carbon peaks (C 1 s) for nPDA powder and sPDA powder respectively. Figure S4, Bacterial adhesion in the medium with 30 min-sPDA-CS and 24 h-nPDA-CS decorated Ti and PEEK. Number of living *E.Coli* and *S.Mutans* in the medium cultured with modified Ti and PEEK after exposed to bacterial suspension for 4 h, 24 h and 72 h. ** represents p<0.01 compared with the pristine group, n = 3.
(DOCX)
