# Supplementary material for: Rapidly-Deposited Polydopamine Coating via High Temperature and Vigorous Stirring: Formation, Characterization and Biofunctional Evaluation
Source: PLoS One. 2014 Nov 21;9(11):e113087. doi: 10.1371/journal.pone.0113087 (PMC4240549; doi:10.1371/journal.pone.0113087)

Combined Supporting Information File S1

Figure S1. Mass change versus time and film thickness versus time for sPDA decorated chips. The black curve shows the mass changes versus time of QCM chips coated by sPDA with various polymerization time (5 min, 10 min, 20 min, 30 min, 1 h, 2 h, 4 h and 8 h) that had ultrasonically cleaned for 10 min. The red curve shows the film thickness versus time of the same samples. n=3.


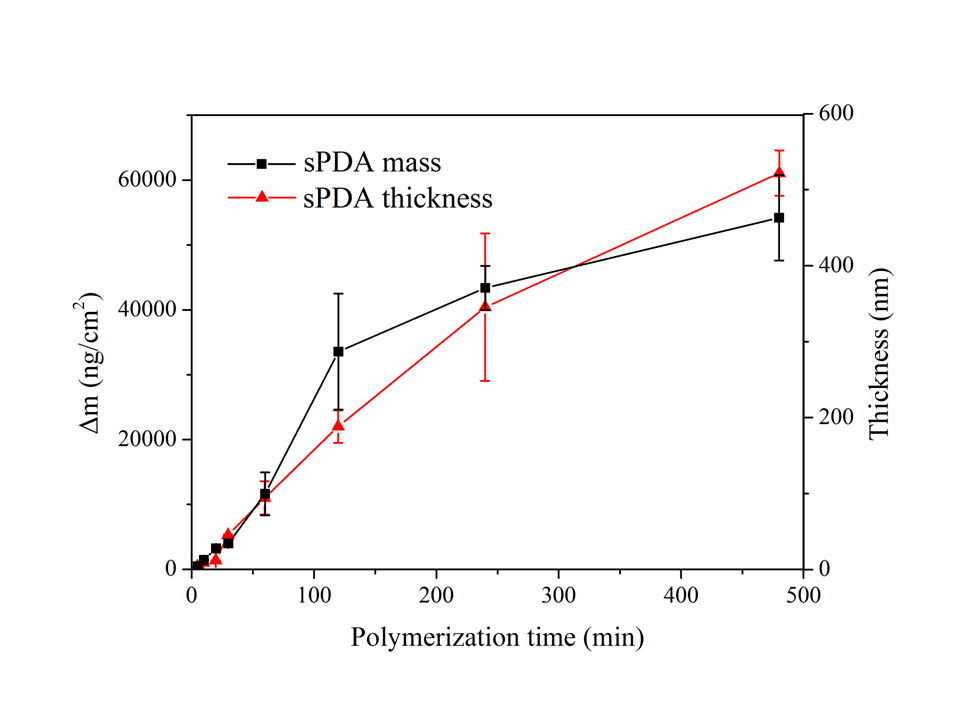


Figure S2. FTIR spectra analysis. The FTIR spectra (KBr) of nPDA powder and sPDA powder.


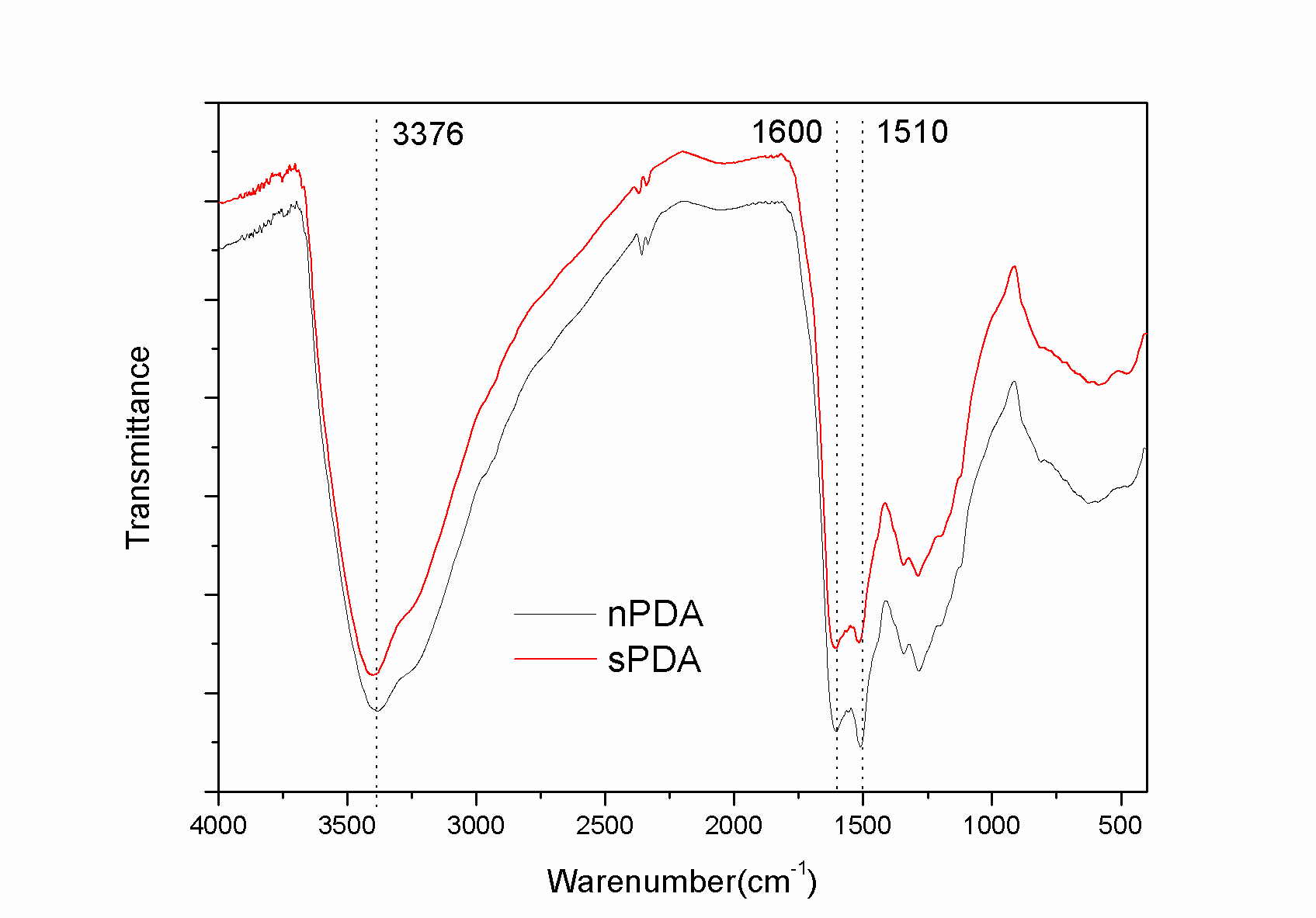


Figure S3. XPS survey scan spectra of nPDA powder and sPDA powder. (A): XPS wide spectra of nPDA powder and sPDA powder, insert table shows the contents of C, N and O. (B): High-resolution spectrum of carbon peaks (C 1s) for nPDA powder and sPDA powder respectively.


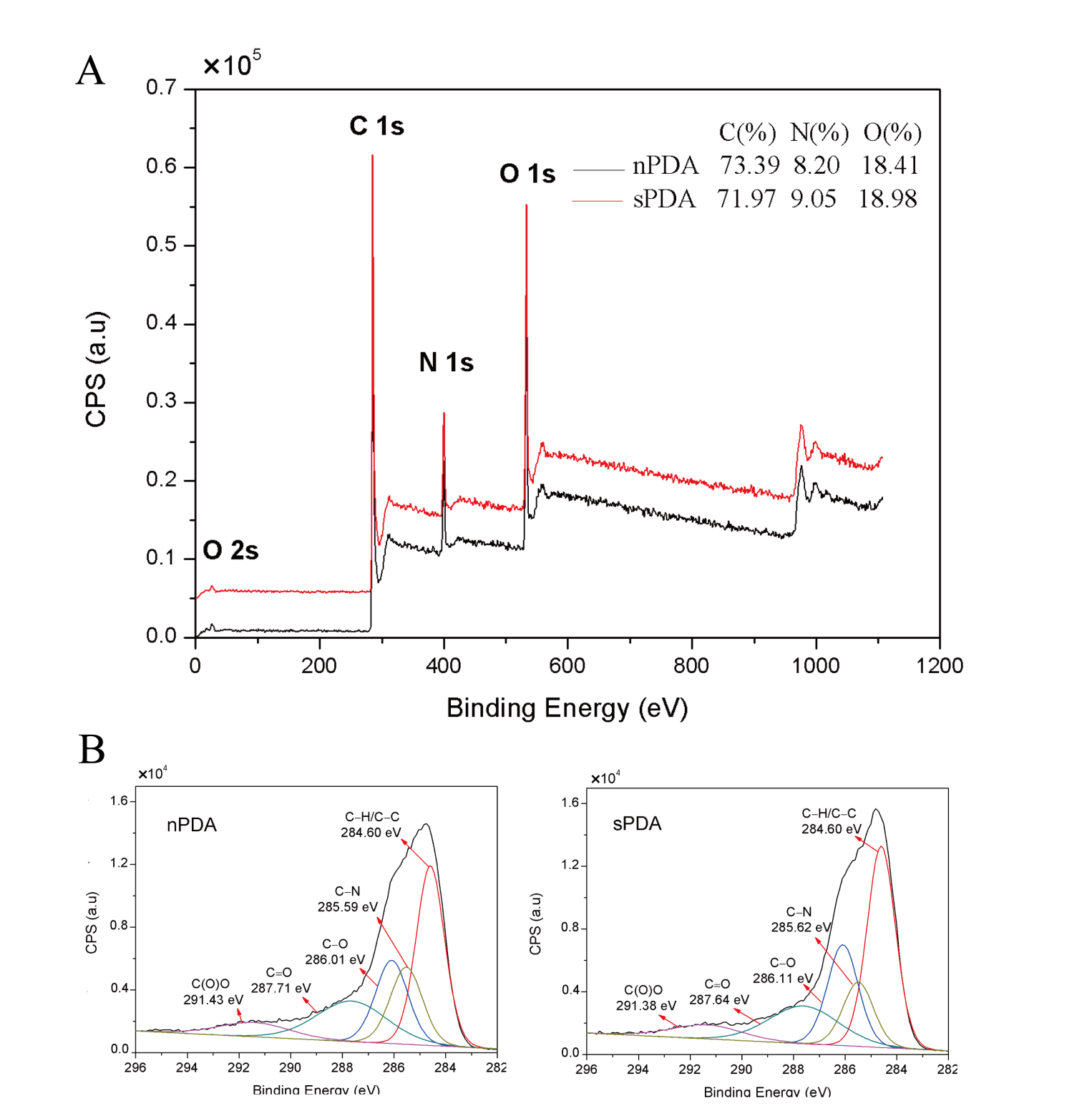


Figure S4. Bacterial adhesion in the medium with 30min-sPDA-CS and 24h-nPDA-CS decorated Ti and PEEK. Number of living *E.Coli* and *S.Mutans* in the medium cultured with modified Ti and PEEK after exposed to bacterial suspension for 4 h, 24 h and 72 h. ** represents p < 0.01 compared with the pristine group, n=3.


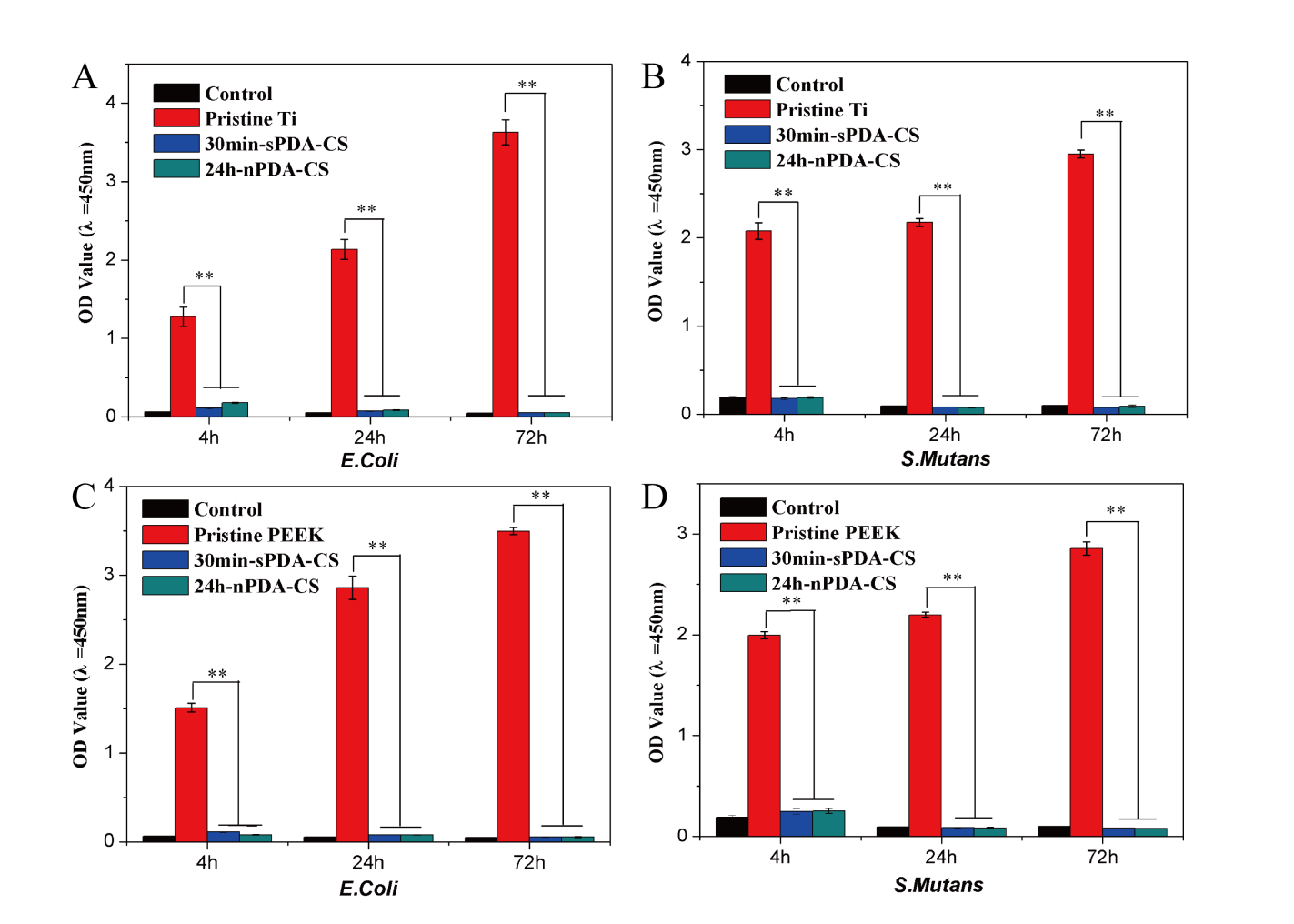

Supplement: File S1 — Contains the following files: Figure S1, Mass change versus time and film thickness versus time for sPDA decorated chips. The black curve shows the mass changes versus time of QCM chips coated by sPDA with various polymerization time (5 min, 10 min, 20 min, 30 min, 1 h, 2 h, 4 h and 8 h) that had ultrasonically cleaned for 10 min. The red curve shows the film thickness versus time of the same samples. n = 3. Figure S2, FTIR spectra analysis. The FTIR spectra (KBr) of nPDA powder and sPDA powder. Figure S3, XPS survey scan spectra of nPDA powder and sPDA powder. (A): XPS wide spectra of nPDA powder and sPDA powder, insert table shows the contents of C, N and O. (B): High-resolution spectrum of carbon peaks (C 1 s) for nPDA powder and sPDA powder respectively. Figure S4, Bacterial adhesion in the medium with 30 min-sPDA-CS and 24 h-nPDA-CS decorated Ti and PEEK. Number of living E.Coli and S.Mutans in the medium cultured with modified Ti and PEEK after exposed to bacterial suspension for 4 h, 24 h and 72 h. ** represents p<0.01 compared with the pristine group, n = 3. (DOCX) [file pone.0113087.s001.docx]
